# Interrelationships between Peak Strain Dispersion, Myocardial Work Indices, Isovolumetric Relaxation and Systolic–Diastolic Coupling in Middle-Aged Healthy Subjects

**DOI:** 10.3390/jcm12175623

**Published:** 2023-08-28

**Authors:** Andrzej Minczykowski, Przemysław Guzik, Anna Sajkowska, Anna Pałasz-Borkowska, Andrzej Wykrętowicz

**Affiliations:** Department of Cardiology-Intensive Therapy, Poznań University of Medical Sciences, 49 Przybyszewskiego, 60-355 Poznań, Poland; pguzik@ptkardio.pl (P.G.); anna_sajkowska@op.pl (A.S.); palasz.anna@wp.pl (A.P.-B.); awykreto@ptkardio.pl (A.W.)

**Keywords:** systolic–diastolic coupling, peak strain dispersion, wasted myocardial work, myocardial work efficiency

## Abstract

In echocardiography, peak strain dispersion (PSD) is the standard deviation of the time to peak longitudinal strain for each left ventricular (LV) segment during systole. It assesses the coordination and synchrony of LV segment contractility. Global work efficiency (GWE) and global wasted work (GWW) quantify LV myocardial work and, if impaired, the coupling between LV systolic contraction and early relaxation. Isovolumetric relaxation (IVRT) measures the duration of initial LV relaxation, while the ratio of early diastolic recoil to systolic excursion (E′_VTI_/S′_VTI_) describes systolic–diastolic coupling. We evaluated these parameters in 69 healthy subjects and found that PSD correlated negatively with GWE (r = −0.49, *p* < 0.0001) and E′_VTI_/S′_VTI_ (r = −0.44, *p* = 0.0002), but positively with GWW (r = 0.4, *p* = 0.0007) and IVRT (r = 0.53, *p* < 0.0001). GWE correlated negatively with GWW (r = −0.94, *p* < 0.0001) and IVRT (r = −0.30, *p* = 0.0127), but positively with E′_VTI_/S′_VTI_ (r = 0.3, *p* = 0.0132). In addition, E′_VTI_/S′_VTI_ was negatively correlated with GWW (r = −0.35, *p* = 0.0032) and IVRT (r = −0.36, *p* = 0.0024). These associations remained significant after adjustment for sex, age and LV mass index of the subjects. In conclusion, there is an interaction between measures of LV asynchrony, myocardial work, diastolic function and its systolic–diastolic coupling in middle-aged healthy subjects. The clinical value of these interactions requires further investigation.

## 1. Introduction

As assessed by speckle-tracking echocardiography (STE), global longitudinal peak systolic strain (GLPSS) can detect early myocardial systolic dysfunction even when the left ventricular ejection fraction (LVEF) remains normal [1]. STE has significant clinical value in various patients, for instance, after myocardial infarction, with heart failure, cardiomyopathies, hypertension or diabetes. Other demonstrated uses of STE include identifying patients at risk of developing heart failure, guiding treatment decisions, assessing heart failure severity, monitoring treatment response and predicting the risk of sudden cardiac death [2,3,4,5,6,7,8].

Peak strain dispersion (PSD) represents the standard deviation of the time-to-peak longitudinal strain for each left ventricular (LV) segment over the entire cardiac cycle [2]. It evaluates how myocardial segment contractility is or is not coordinated and synchronized throughout LV systole. Higher PSD values show larger variability and worse contractile synchronicity of various LV segments. PSD is increased in patients with diabetes or hypertension, after myocardial infarction, regardless of the normal GLPSS and LVEF [9,10]. Its clinical utility has also been demonstrated in patients following myocardial infarction and heart failure. PSD may help to identify the severity of heart failure and predict the risk of death or hospitalization [11,12].

LV pressure–strain loops are used to assess the myocardium by several parameters. Global work efficiency (GWE) and global wasted work (GWW) are two commonly used examples [1,13,14,15]. Myocardial work indices also quantify LV systolic function and its abnormalities and reflect global LV systolic function [1,13,14]. However, GWE and GWW are sensitive to late systolic dysfunction due to delayed contraction of some LV segments. Such LV segments do not contribute to blood ejection into the aorta as they continue to contract after aortic valve closure in the early phases of LV diastole. As GWE and GWW depend on phenomena occurring during systole and diastole, both indices are markers of the temporal bridging and interaction between both phases of the cardiac cycle.

Abnormal GWW and GWE have been reported in cardiac patients [15,16,17,18,19], for instance, an additional prognostic value of myocardial work parameters following acute myocardial infarction. Lower GWE at 1 month after myocardial infarction was independently associated with higher major event rates. At the same time, GWE < 91% identified patients at higher risk of myocardial infarction. GWW and GWE were correlated with age, body mass index, systolic blood pressure, smoking history and left ventricular ejection fraction (LVEF) in diabetic patients [20].

Afterload is the pressure the LV must exert to eject blood during contraction. Changes in afterload affect stroke volume and end-systolic and end-diastolic volumes and pressures. The LV isovolumetric relaxation time (IVRT) is the first LV diastolic parameter that afterload may modify [21,22,23]. Both PSD and myocardial work indices are time-dependent. Delayed contractility of certain LV segments should increase PSD and GWW and reduce GWE. As they cover the transitory zone between systole and diastole, i.e., mostly isometric relaxation, both indices might quantify the coupling between LV systolic contraction and the earliest diastole phase.

Tissue Doppler measures tissue velocities of LV segments in different places, for example, at the level of septal and lateral parts of the mitral annulus during systole and diastole. The velocity time integral for the systolic part (S′_VTI_) reflects the total upward movement of LV segments toward the apex. In contrast, the velocity integral of the early diastole (E′_VTI_) quantifies the total downward movement during the early LV filling. The E′_VTI_/S′_VTI_ ratio is considered an index of LV systolic–diastolic coupling.

LV compliance reflects how easily this ventricle stretches in response to blood filling during early diastole. The nadir of early diastolic tissue Doppler velocity (E′) represents the velocity at which the mitral annulus moves downward during early diastole and how the mitral annulus relaxes. The peak value of the early diastolic mitral valve flow velocity (E) by pulsed-wave Doppler shows the early filling of the LV by blood from the left atrium. The E/E′ ratio is an approximation of the LV filling pressure and thus LV compliance.

The Frank–Starling principle states that the extent to which ventricles are stretched with blood during diastole is related to the force of contraction during systole [24]. In other words, the diastolic function determines systolic performance. Therefore, changes in filling pressure and time, relaxation rate and time, systolic–diastolic coupling and ventricular compliance affect stroke volume and myocardial work. On the other hand, poorer systolic function affects LV diastolic indices, e.g., impaired contractility is associated with poorer compliance, higher filling pressures, etc. Both systole and diastole are important for proper cardiac function.

PSD and GWE are mainly associated with LV systole. GWW can include both LV systole and early diastole. IVRT represents early diastole, while E′_VTI_/S′_VTI_ combines systole and early diastole beyond IVRT. Mutual associations among these parameters are uncertain. To address this issue, we designed this study to investigate peak strain dispersion in relation to myocardial work indices, IVRT and systolic–diastolic performance in a group of healthy people.

## 2. Materials and Methods

For the study, we used a set of rules for participants’ screening, selection and enrollment. To identify healthy participants, we used the following criteria:

- No known acute or chronic illness—participants were required to have no documented history of acute or chronic illness;
- Absence of signs and symptoms of acute disease—individuals were assessed for any physical signs or complaints related to underlying disease;
- No chronic medication use, except for oral hormonal contraception in women of reproductive age;
- Smokers were allowed to participate in the study;
- Occasional use of nonsteroid anti-inflammatory drugs for occasional pain relief (e.g., headache) was allowed, except for 48 h before ECG and echocardiography.

All potential participants underwent a comprehensive clinical evaluation consisting of the following components:

- Brachial blood pressure;
- Standard 12-lead ECG recordings were obtained to assess the presence of sinus rhythm and to detect any abnormal findings;
- Transthoracic 2D echocardiography using a GE Vivid E95 platform.

Only good-quality images and cine loops were accepted to accurately measure left ventricular longitudinal strain and myocardial work (MW) during postprocessing. In addition, the following technical requirements were mandatory for all healthy participants: no myocardial contractile abnormalities or clinically significant valvular disease. Participants were not eligible if they had valvular disease with more than mild regurgitation or stenosis. Anthropometric measurements (height, weight) were all made according to standard protocol. Sixty-nine healthy adult volunteers fulfilled all enrollment criteria. All presented sinus rhythm and normal findings on ECG. Brachial blood pressure was acquired by an oscillometric method (705IT, Omron Healthcare Co., Ltd., Kyoto, Japan) in the supine position (mean of three measurements) after 10 min of rest and was <130/80 mmHg.

The University Ethics Committee approved the study protocol. Informed consent was obtained from all participants. The Declaration of Helsinki was followed in the study [25].

### 2.1. Echocardiography

Transthoracic echocardiography using a commercially available ultrasound system (Vivid E95, GE Healthcare, Horten, Norway) was performed at rest with the subjects in the left lateral recumbent position. All procedures were performed using standard views according to the recommendations of the American Society of Echocardiography [26]. Digital images were stored and transferred to a computer workstation (EchoPAC version 202, GE Healthcare, Horten, Norway) for further offline analysis. Measurements of cardiac chamber dimensions, volumes and wall thickness were collected during diastole and systole. According to the modified Simpson’s rule, LV volumes and LVEF were estimated as the mean value from apical four- and two-chamber views.

The peak value of the mitral valve early diastolic flow velocity (E) was measured with pulsed-wave Doppler. Tissue Doppler was used to obtain the septal and lateral mitral annular systolic (S′) and early diastolic velocities (E′). Velocity time integrals were measured by tracing the systolic signal to measure mitral annulus systolic excursion (S′_VTI_) and the early diastolic signal to measure early diastolic excursion (E′_VTI_). These values were then used to calculate average S′ and E′ velocities, E/E′ ratio and E′_VTI_/S′_VTI_ ratio [27]. IVRT was also measured as the interval between the end of S′ and the beginning of E′ from the same tissue Doppler signal. Measurements were made separately for the septal and lateral segments and then averaged.

Continuous dynamic images optimized for speckle tracking analysis were obtained at apical four-, three- and two-chamber views at a frame rate of at least 60 frames/s. The images were imported into the software dedicated for analysis (EchoPAC version 202). Automatic function imaging was used to automatically track each endocardial and epicardial boundary in the three apical dynamic images, and the region of interest (ROI) was adjusted by correcting the endocardial border or width, if necessary. Peak systolic longitudinal strain was estimated in 6 segments per one view, giving 18 values per patient. The peak strain dispersion (PSD), a synchronization marker of the myocardial contraction in the left ventricle, was calculated. Global longitudinal peak systolic strain (GLPSS) was achieved by averaging over 18 segmental peak systolic longitudinal strain values. Brachial artery pressure was measured before the echocardiographic examination, and GLPSS were used to quantify global LV myocardial work efficiency through the noninvasive LV pressure–strain analysis [15,18,19,28]. In this method, the automated function imaging software creates the LV pressure–strain loop curve adjusted to the duration of isovolumic and ejection phases, defined by mitral and aortic valvular timing events evaluated through 2D echocardiography. The area within the LV pressure–strain loop is used as an index of global myocardial work (GWI). Related indices were then calculated: global constructive work (GCW), an effective work performed during the systolic shortening and isovolumic diastolic myocardial elongation; global wasted work (GWW), which is classified as work not conducted for LV ejection, including systolic myocardial stretching during systole and isovolumic diastolic period shortening; and global myocardial work efficiency (GWE), calculated as the percentage ratio of CGW to the sum of CGW and GWW.

For this study, only one cardiologist (A.M.) experienced in clinical echocardiography acquired all echocardiographic images and cine loops, and performed all postprocessing analyses using EchoPAC. We have tested his intrarater variability for tissue Doppler, GLPSS, MW and other measures, and the observed variability was in the range 3–6%, demonstrating the consistency of the measurements performed.

**Figure 1:**
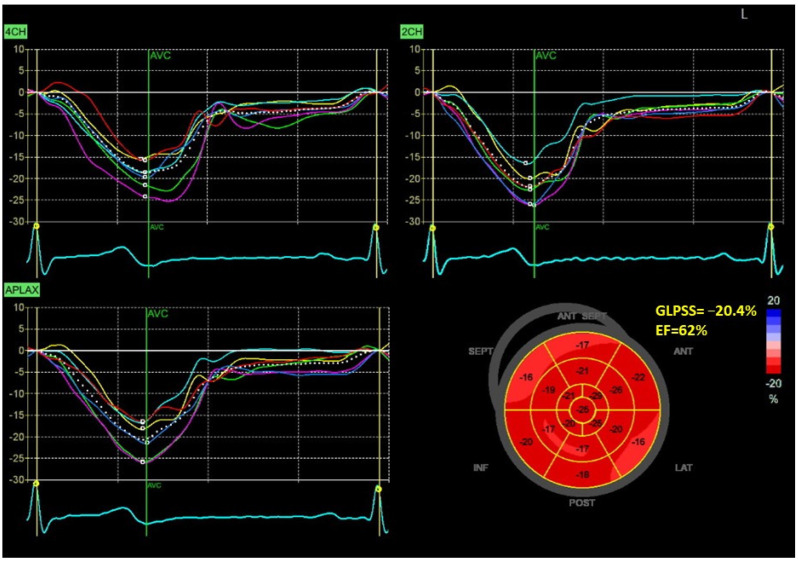

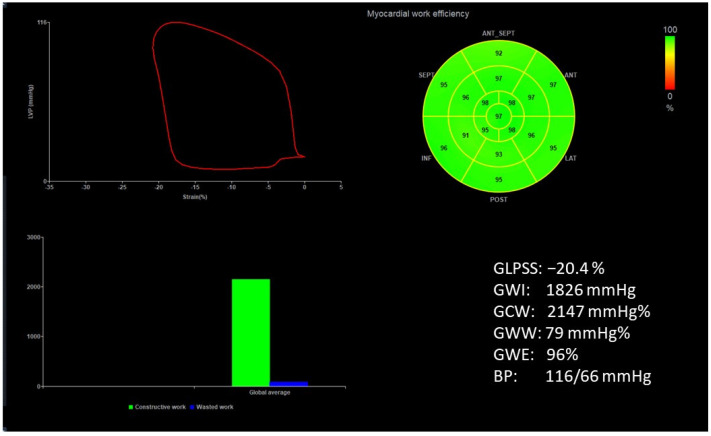
An example of echocardiography of a 30-year-old healthy male. A set of individual longitudinal strain curves for all segments shown in different colors, with the average curve shown as a white dotted line, a bull’s eye of segmental longitudinal peak systolic strains and a summary of global longitudinal peak systolic strain (GLPSS) are shown in the top panel. The LV pressure–strain loop with the bull’s eye representation of myocardial work efficiency for each of the segments and a summary of all myocardial work indices are shown in the lower panel. Abbreviations: GLPSS—global longitudinal peak systolic strain, EF—ejection fraction, GWI—global myocardial work index, GCW—global constructive work, GWW—global wasted work, GWE—global work efficiency, BP—blood pressure.

**Figure 2:**
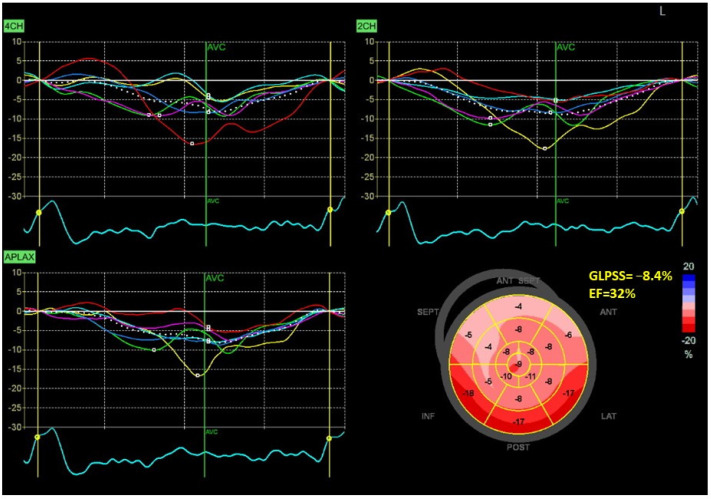

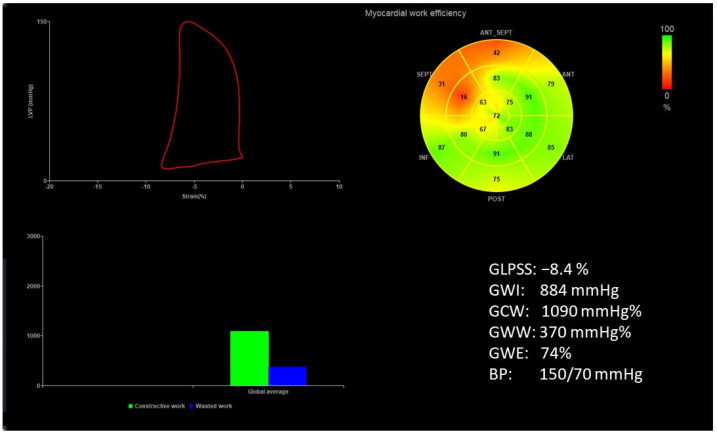
An example of echocardiography of a 61-year-old man with the left bundle branch block and a history of myocardial infarction. A set of individual longitudinal strain curves for all segments shown in different colors, with the average curve shown as a white dotted line, a bull’s eye of segmental longitudinal peak systolic strains and a summary of global longitudinal peak systolic strain (GLPSS) are shown in the top panel. The LV pressure–strain loop with the bull’s eye representation of myocardial work efficiency for each of the segments and a summary of all myocardial work indices are shown in the lower panel. Abbreviations: GLS—global longitudinal peak systolic strain, EF—ejection fraction, GWI—global myocardial work index, GCW—global constructive work, GWW—global wasted work, GWE—global work efficiency, BP—blood pressure.

Figure 1 and Figure 2 show two contrasting examples (a healthy person and a patient after myocardial infarction and left bundle branch block) of STE curves with GLPSS, LVEF and LV pressure–strain loops and results of GWE for all LV segments.

### 2.2. Statistical Analysis

Continuous data had a normal distribution (according to the D’Agostino–Pearson normality test); results are summarized as mean and standard deviation (SD) [29]. Numbers and percentages were used for categorical variables. Pearson correlation was first used to examine the association between peak strain dispersion, myocardial work indices, isovolumetric relaxation and systolic–diastolic coupling of peak strain dispersion. These reciprocal associations were then examined using linear regression models adjusted for sex, age and LVMI. All tests were two-sided. The statistical analyses and graphs were made using JMP Pro 17 (SAS Institute Inc., Cary, NC, USA), and the statistical significance was set at *p* < 0.05.

## 3. Results

Sixty-nine healthy subjects were examined (mean age 58 ± 6 years, 39 females). Essential clinical and echocardiographic characteristics are presented in Table 1. Study subjects revealed an average ejection fraction (62 ± 5%), global longitudinal peak systolic strain (GLPSS) of −18 ± 2%, global work efficiency of 94 ± 3%, and peak strain dispersion of 44 ± 12 ms.

The Interrelations between Descriptors of Strain Dispersion, Myocardial Work, Isovolumetric Relaxation and Systolic–Diastolic Coupling

Table 2 summarizes PSD, GWE, GWE, IVRT and E′_VTI_/S′_VTI_ correlations. PSD correlated negatively with GWE and E′_VTI_/S′_VTI_ but positively with GWW and IVRT. GWE correlated negatively with GWW (nearly linearly) and IVRT but positively with E′_VTI_/S′_VTI_. Negative correlations were found between E′_VTI_/S′_VTI_ and GWW or IVRT.

**Figure 3:**
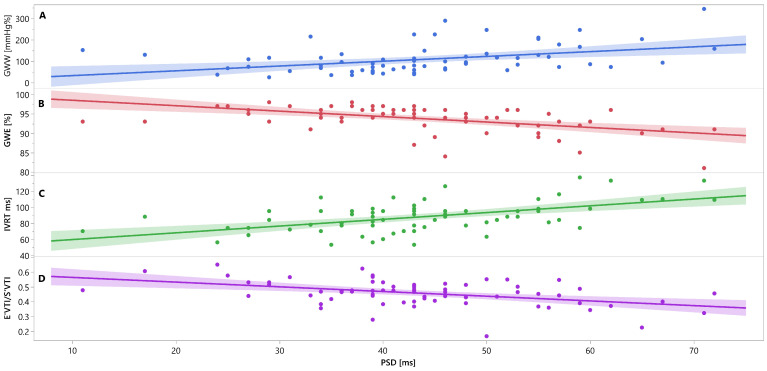
Linear regression lines for associations between PSD and GWW (panel A, line and points in blue), GWE (panel B, line and points in red), IVRT (panel C, line and points in green), and E′_VTI_/S′_VTI_ (panel D, line and points in purplein healthy people. Abbreviations: E′_VTI_/S′_VTI_—systolic–diastolic coupling, GWW—global wasted work, GWE—global work efficiency, IVRT—isovolumetric relaxation time, PSD—peak systolic dispersion.

**Figure 4:**
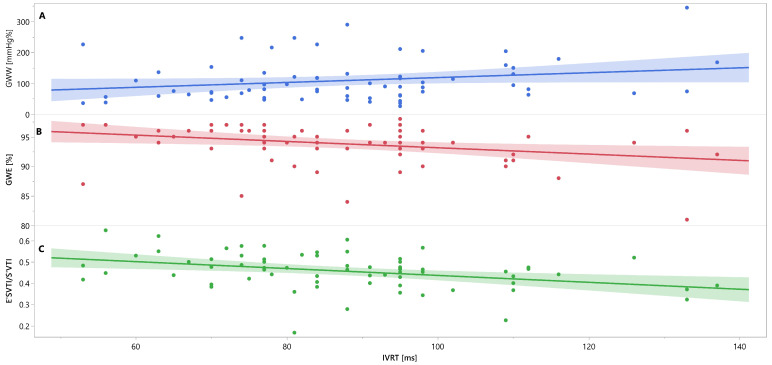
Linear regression lines for associations between IVRT and GWW (panel A, line and points in blue), GWE (panel B, line and points in red), and E′_VTI_/S′_VTI_ (panel C, line and points in green) in healthy people. Abbreviations: E′_VTI_/S′_VTI_—systolic–diastolic coupling, GWW—global wasted work, GWE—global work efficiency, IVRT—isovolumetric relaxation time.

**Figure 5:**
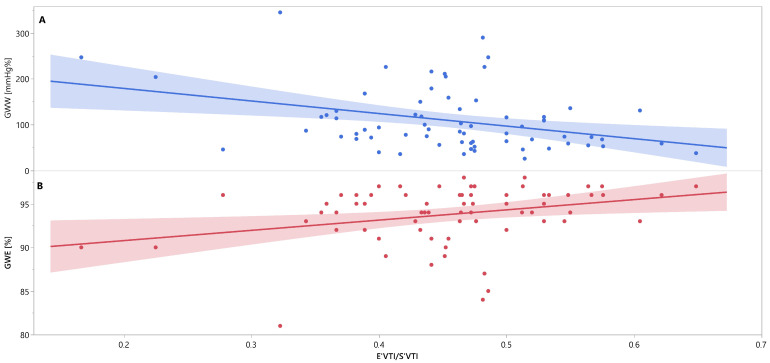
Linear regression lines for associations between E′_VTI_/S′_VTI_ and GWW (panel A, line and points in blue), and GWE (panel B, line and points in red) in healthy people. Abbreviations: E′_VTI_/S′_VTI_—systolic–diastolic coupling, GWW—global wasted work, GWE—global work efficiency.

Mutual associations between PSD, GWW, GWE, IVRT and E′_VTI_/S′_VTI_ are additionally shown in Figure 3, Figure 4 and Figure 5 as linear regressions.

Irrespective of participants’ sex, age and LVMI, most reciprocal associations remained statistically significant, as did the Pearson correlation (Table 3.). There was no change in the direction of these associations—negative remained negative, and positive retained positive. Only the associations between IVRT and GWE (or vice versa) became not quite statistically significant (*p* = 0.0611). The associations between IVRT and GWW were still insignificant for the Pearson correlation.

## 4. Discussion

The study found that indices of mechanical dispersion (PSD), myocardial work (GWE and GWW), isovolumetric relaxation (IVRT) and systolic–diastolic coupling (E′_VTI_/S′_VTI_) are interrelated in middle-aged healthy subjects. Specifically, worse systolic function (higher PSD and GWW but lower GWE) is associated with longer IVRT and lower E′_VTI_/S′_VTI_. It may imply an impaired early diastolic function and ventricular–arterial coupling.

Higher PSD represents increased heterogeneity of LV contraction and is associated with worse GWE, systolic–diastolic coupling (E′_VTI_/S′_VTI_), increased GWW and longer IVRT. GWE and GWW are inversely correlated, which is not surprising. The wasted myocardial work increases due to worsened work efficiency. Looking further ahead, longer IVRT translates into an impaired ability of the LV to relax and prepare to be filled with blood from the left atrium. Interestingly, worse GWE and E′_VTI_/S′_VTI_ are associated with longer IVRT. Finally, E′_VTI_/S′_VTI_ is impaired when PSD, GWW and IVR increase. Except for the association of IVRT with GWE and GWW, all the associations studied are independent of sex, age and LVMI of healthy subjects.

We conducted the study in healthy people, and the measurements were within normal ranges. Therefore, the diagnosis of systolic and diastolic dysfunction may be inappropriate. Instead, we suggest that if a person has a systolic function that is at the lower end of the normal range, then the diastolic function should also be expected to be reduced.

It was recently shown that in a subgroup of well-controlled diabetic patients, GLPSS was similar to healthy controls, whereas PSD was significantly higher in diabetic patients [9]. Similar findings have been reported in patients with uncomplicated systemic lupus, namely higher PSD and normal GLPSS compared to healthy controls [30]. Surprisingly, there are only sparse data on peak systolic strain dispersion and its correlates in healthy subjects.

PSD reflects whether the peak time of the long-axis strain of the LV myocardium is consistent and more or less uniform throughout all contracting segments. Therefore, a higher PSD translated to LV dyssynchrony. This parameter indicates an early stage of systolic dysfunction in diabetes and hypertension, which may not be detected by other markers such as ejection fraction [9,10]. Seo et al. [31] showed that LVMI was independently associated with LV dyssynchrony, measured as the standard deviation of the mean time-to-peak systolic velocity of 12 middle and basal left ventricular segments.

LV dyssynchrony results in inefficient LV contraction and reduced cardiac output. Myocardial work indices describe the productive and unproductive work of LV segments, i.e., those that contribute to ejection and those that do not. We show that GWW is positively correlated with PSD. In contrast, both PSD and GWW are negatively associated with GWE. These observations suggest that unsynchronized contraction of LV segments, represented by increased PSD, contributes to GWW and is responsible for worsening GWE.

A close relationship between systolic contraction and relaxation (systolic–diastolic coupling) influences global LV performance. E′_VTI_/S′_VTI_, a measure of systolic–diastolic coupling, describes the ratio of early diastolic recoil to systolic excursion, as explained by MacNamara et al. [27] with the spring model of LV longitudinal motion. In other words, it shows which part of the systolic movement of the LV base toward the apex is compensated during early diastole. Heart failure patients with preserved LVEF and comparable E′/E or E′ had reduced E′_VTI_/S′_VTI_ compared to healthy subjects.

We show that E′_VTI_/S′_VTI_ worsens when both systolic and diastolic function decline (negative correlations with PSD, GWW and IVRT, and positive with GWE). Notably, these associations were still significant after adjustment for sex, age and LVMI of the subjects.

Assessing mechanical dispersion, myocardial work, isovolumetric relaxation and systolic–diastolic coupling can provide complementary information on the global and regional LV function and performance in healthy subjects. All of these parameters have demonstrated clinical value [1,11,12,13,14,15,16,17,18,19,20,27,32,33,34,35,36,37,38,39,40].

### 4.1. Study’s Limitations

It is important to mention the study’s limitations, which must be addressed in future research. First, it is a medium-sized study conducted only on healthy middle-aged people. Therefore, the results may not apply to other age groups. Second, extrapolating our findings to patients with different diseases, risk factors, conditions and complications may not be justified. Third, this was an observational physiological study. It takes more than observed correlations between different parameters to explain all the physiological mechanisms behind them. We are aware that correlations do not imply causation. A final limitation relates to speckle tracking echocardiography (STE) for analyzing PSD and myocardial work indices.

PSD and GWE or GWW are derived from STE and are based on identifying LV deformation in the longitudinal direction. STE is mainly used to quantify LV systolic function. For GLPSS, the relative change in myocardial length from end-diastole to end-systole is assessed. Any problems in obtaining good quality STE automatically carry over to measurements of PSD and myocardial work indices. Some examples are imperfect tracking of myocardial motion in areas of low echogenicity. This can lead to inaccurate strain measurements in these areas.

Another limiting factor for STE is the dependence on the imaging plane. The myocardium is not a homogeneous structure, and strains can vary in different regions of the myocardium. For STE, and thus PSD, GWE and GWW, strain measurements are typically made in the apical four-, two- and three-chamber views. Although three planes are commonly used, they are not equivalent to real three-dimensional signal acquisition and strain. Some mathematical approximations are introduced that may affect the final results. Also, any inaccuracies in the plane images will affect the final measurement.

Occasionally, it is not possible to obtain good quality images in all three planes—this is often the case in patients with complex cardiac anatomy, obesity, narrow intercostal spaces or atypical heart position in the chest. Other limiting factors associated with STE are more technical, such as the frame rate of the acquired signal, the signal-to-noise ratio, angle dependence and intervendor variability. These factors can limit the accuracy and reproducibility of STE and, therefore, PSD and myocardial work analysis.

Unfortunately, strain measurements are not standardized across different software platforms, which may lead to inconsistent results and interpretation. In our study, we used the same echocardiographic system and performed all measurements using the same postprocessing platform. However, differences between echo system vendors may limit the applicability and generalizability of STE-based findings.

Despite these limitations, STE is a valuable tool for assessing LV function. The technology continues to improve, and PSD and myocardial work are good examples of this progress. Furthermore, many clinical studies have provided evidence for their practical usefulness in all these measures.

### 4.2. Potential Clinical Implications and Perspectives

We provide new insights into the relationships between mechanical dispersion, myocardial work, isovolumetric relaxation and systolic–diastolic coupling in healthy subjects. These parameters reflect different LV function and performance aspects, such as LV synchrony, efficiency, diastolic relaxation and systolic–diastolic interaction.

Investigation of these relationships may be helpful to understand better the physiology and clinical findings of various cardiac diseases that affect LV function and performance, such as ischemic heart disease, heart failure, cardiomyopathies, valvular heart disease, diabetes, obesity, hypertension or atrial fibrillation. Studying these relationships can also help assess the effects of any drug that directly or indirectly affects the myocardium and cardiac function. Such drugs include typical cardiac medications for hypertension or heart failure and other drugs such as chemotherapy with cardiotoxic effects or antipsychotic/antidepressant treatment with cardiac side effects.

The relationships described can also be studied in patients undergoing cardiac surgery, before and after implantation of cardiac devices, treatment of valvular heart disease and nonpharmacological interventions. Many treatment modalities have an impact on LV function and performance. For example, interventions that correct regional wall motion abnormalities may also improve myocardial work efficiency and reduce wasted work. Implanted cardiac devices optimizing LV synchrony may also improve IVRT and E′_VTI_/S′_VTI_.

Nonpharmacological interventions that modify lifestyle or risk factors, such as dietary intervention and increasing physical activity, may also have beneficial effects on PSD, myocardial work, diastolic function, etc. Therefore, these parameters may serve as noninvasive and inexpensive echocardiographic biomarkers to monitor the response and outcome of various therapeutic modalities in patients with impaired LV function and performance.

## 5. Conclusions

Overall, there is interaction between measures of LV asynchrony, myocardial work, diastolic function and its systolic–diastolic coupling in middle-aged healthy individuals. These parameters provide complementary information on global and regional LV function and performance. How these interactions change in different clinical conditions and whether any treatment can modify them is unknown and deserves further study.

## Figures and Tables

**Table 1:** Clinical and echocardiographical characteristics of study subjects.

Characteristics	
Age (years)	58 ± 6
Female/male	39/30
BP systolic (mmHg)	122 ± 15
BMI (kg/m^2^)	26 ± 4
IVSd (mm)	11 ± 2
LVPWd (mm)	10 ± 1
LVEDd (mm)	43 ± 5
LVEDs (mm)	28 ± 5
LA (mm)	33 ± 4
LVMI (g/m^2^)	87 ± 19
LVEDV (mL)	94 ± 27
LVESV (mL)	36 ± 14
SV (mL)	55 ± 15
CO (L/min)	4.0 ± 1.1
EF (%)	63 ± 5
E/A	1.1 ± 0.3
E/E′	7 ± 2
GLPSS (%)	−18 ± 2
IVRT (ms)	87.8 ± 18.8
PSD (ms)	44 ± 12
GWE (%)	94 ± 3
GWW (mmHg%)	109 ± 67
E′_VTI_/S′_VTI_	0.46 ± 0.1

Abbreviations: BP—blood pressure, BMI—body mass index, IVSd—end-diastolic thickness of the intraventricular septum, LVPWd—end-diastolic thickness of the left ventricular posterior wall, LVEDd—left ventricular end-diastolic diameter, LVEDs—left ventricular end-systolic diameter, LA—left atrial diameter, LVMI—left ventricular mass index, LVEDV—left ventricular end-diastolic volume, LVESV—left ventricular end-systolic volume, SV—stroke volume, CO—cardiac output, EF—ejection fraction, E/A—E to A waves ratio, E/E′—E to E′ ratio, GLPSS—global longitudinal peak systolic strain, IVRT—isovolumetric relaxation time, PSD—peak strain dispersion, GWE—global work efficiency, GWW—global wasted work, E′_VTI_/S′_VTI_—systolic–diastolic coupling.

**Table 2:** The r coefficients of Pearson’s correlation between PSD, GWW, GWE, IVRT and E′_VTI_/S′_VTI_ in healthy people. Neither repeated nor identical parameter correlations (X) are shown.

Clinical Variable	GWE	GWW	IVRT	E′_VTI_/S′_VTI_
PSD	−0.49; *p* < 0.0001	0.40; *p* = 0.0007	0.53; *p* < 0.0001	−0.44; *p* = 0.0002
GWE	X	−0.94; *p* < 0.0001	−0.30; *p* = 0.0127	0.30; *p* = 0.0132
GWW		X	0.22; *p* = 0.0661	−0.35; *p* = 0.0032
IVRT			X	−0.36; *p* = 0.0024

Abbreviations: E′_VTI_/S′_VTI_—systolic–diastolic coupling, GWW—global wasted work, GWE—global work efficiency, IVRT—isovolumetric relaxation time, PSD—peak systolic dispersion, X—correlations with r = 1 for the same parameters, e.g., PSD with PSD.

**Table 3:** Linear regression models adjusted for participants’ gender, age and left ventricular mass index showing beta coefficients (slopes of regression lines) and *p* values for the reciprocal associations between PSD, GWW, GWE, IVRT and E′_VTI_/S′_VTI_ in healthy people. Independent variables are presented in columns and dependent variables in rows. X represents cases where the same parameter cannot be both a dependent and an independent variable.

		Independent variable				
		PSD	GWE	GWW	IVRT	E′_VTI_/S′_VTI_
Dependent variable	PSD	X	−1.3421; *p* = 0.0002	0.053; *p* = 0.004	0.24; *p* = 0.0003	−45.1867; *p* = 0.0042
	GWE	−0.1471; *p* = 0.0002	X	−0.0473; *p* = <0.001	−0.0430; *p* = 0.0611	10.9539; *p* = 0.0391
	GWW	2.3052; *p* = 0.004	−18.7845; *p* = <0.0001	X	0.5842; *p* = 0.2055	−267.604; *p* = 0.0107
	IVRT	0.7626; *p* = 0.0003	−1.2481; *p* = 0.0611	0.0427; *p* = 0.2055	X	−64.0973; *p* = 0.0245
	E′_VTI_/S′_VTI_	−0.0027; *p* = 0.0042	0.0059; *p* = 0.0391	−0.0004; *p* = 0.0107	−0.0012; *p* = 0.0245	X

Abbreviations: E′_VTI_/S′_VTI_—systolic–diastolic coupling, GWW—global wasted work, GWE—global work efficiency, IVRT—isovolumetric relaxation time, PSD—peak systolic dispersion.

## Data Availability

Not applicable.
